# Habitual violent media exposure does not bias facial emotional processing: a comparison of interactive vs. non-interactive content

**DOI:** 10.1038/s41598-025-26041-w

**Published:** 2025-11-26

**Authors:** Anantha Ubaradka, Sanjram Premjit Khanganba

**Affiliations:** 1 Human Factors & Applied Cognition Lab, Indian Institute of Technology Indore, Indore, 453552 India; 2 Discipline of Psychology, Indian Institute of Technology Indore, Indore, 453552 India; 3 Center for Electric Vehicles and Intelligent Transport Systems, Indian Institute of Technology Indore, Indore, 453552 India; 4 Department of Biosciences and Biomedical Engineering, Indian Institute of Technology Indore, Indore, 453552 India; 5 Center of Futuristic Defense and Space Technologies, Indian Institute of Technology Indore, Indore, 453552 India

**Keywords:** Violent media exposure, Aggression, Happy-face advantage, General aggression model, Emotion recognition, Media interactivity, Neuroscience, Psychology, Psychology

## Abstract

The relationship between violent media exposure and aggression remains widely debated. The General Aggression Model (GAM) suggests that repeated exposure fosters aggression through the formation of aggressive scripts and emotional desensitization. However, empirical findings have been mixed, and a growing body of research has criticized the GAM for overstating the strength and consistency of this association. In light of this, the present study investigated whether habitual exposure to violent media biases emotional information processing and whether such effects differ by mode of media interactivity. A total of 54 violent media users participated, comprising violent video gamers (VVGs; *M*_*age*_ = 20.07 years, *SD* = 1.26) and non-video gamers (NVGs; *M*_*age*_ = 21.29 years, *SD* = 1.10). Results showed a robust happy-face advantage and lower recognition capacity for negative emotions across both groups. These findings challenge the script theory and desensitization assumptions. Interestingly, when trait aggression was included as a covariate in the ANCOVA, the emotional effects were consistently attenuated or rendered nonsignificant. This pattern suggests that media effects are shaped by personal predispositions rather than acting in isolation. While the findings challenge the claim that violent media increases aggression, caution is needed when generalizing to individuals with higher levels of dispositional aggression.

**Supplementary Information:**

The online version contains supplementary material available at 10.1038/s41598-025-26041-w.

## Introduction

Violence in digital media is a common phenomenon, often portrayed through characters engaging in physical harm toward others on screen^1^. The presence of virtual hostility is primarily intended to generate thrill and excitement in the audience. While such depictions are accepted as part of mainstream media, they have also raised concerns about the potential increase in aggression and subsequent violent behavior. As a result, the prevalence of violent media has become a focal point of debate within academia and the general public, prompting extensive inquiry into its possible effects on users. Additionally, if violent media does increase aggression, a critical question arises as to whether the impact varies depending on the mode of media interaction.

Interactive formats such as video games enable users to directly participate in the aggressive content^2^. Here, the course of violence and the outcomes are controlled by the player. Conversely, non-interactive formats like films, television, and documentaries position the user as a passive spectator, observing rather than enacting aggression^3^. There are two competing perspectives on the relative impact of interactive and non-interactive violent media content on aggression. One line of argument posits that violent video games would have modest effects on aggression compared to passive media consumption^4,5^. Proponents of this view highlight several factors, such as the unrealistic graphics, the abstract nature of the violence, and the non-human characters typically found in video games^4^. However, as video games have become increasingly realistic, these arguments require re-evaluation. Contemporary video games now feature highly immersive and lifelike content, blurring the line between virtual and real-world experiences^6^. This shift has fueled an argument that video games could exert a stronger influence on aggression than passive viewing^7^. Video games require players to actively engage in violent acts, make decisions, and assume the roles of characters within the gameplay^8^. The active involvement leads to a more direct rehearsal of violent behaviors, potentially increasing the likelihood of players internalizing and acting upon the aggressive behaviors they experience in the gameplay^7^.

Nonetheless, before drawing conclusions about the role of media interactivity, it is essential to examine whether there exists a meaningful association between violent media exposure and aggression itself. The General Aggression Model (GAM) has been the most widely used framework for explaining this relationship^9^. Despite its widespread adoption, the model has faced considerable backlash, with many scholars challenging its empirical validity and generalizability^10,11^. Critics have highlighted issues such as publication bias and the tendency of GAM-based studies to report relatively weak and inconsistent effect sizes when linking violent media to aggression^12–15^. Given this ongoing debate, the present study aims to address two key research problems. First, it examines whether violent media exposure increases aggression or if the concerns outlined by the model are overstated. While the GAM explains aggression through three broad internal states, this study specifically focuses on the effect of violent media on emotional processing. Second, suppose there happens to be an association between violent media exposure and aggression. In that case, the study investigates whether this relationship differs based on the mode of media interaction, comparing interactive (e.g., video games) and non-interactive (e.g., films) modalities.

### Effects of violent media exposure on emotional processing

The GAM adopts a “person-in-situation” approach, conceptualizing aggression as a product of immediate social interactions and long-term personality development^9,16–18^. Its single-episode cycle explains which thoughts and emotions are primed and, therefore, shape the immediate appraisal and subsequent decision-making process^9^. According to this perspective, violent media exposure strengthens “hostile knowledge structures” (e.g., aggressive scripts and schemata), making them chronically accessible and thus more likely to be activated during the cycle’s impulsive appraisal stage^17^. When these knowledge structures are primed, the ambiguous cues are interpreted as hostile, producing a “hostile attribution bias” that elevates anger and readies aggressive behavior^9^. Even a brief exposure to violent media is found to increase the accessibility of aggressive thoughts^19^, while habitual exposure predicts stronger hostile attribution bias^20,21^. Such biased information processing is theorized to impair affective functioning, particularly in the recognition and interpretation of emotional expressions.

Early studies employed the “Emotional Stroop” task to investigate this phenomenon, in which attentional allocation is assessed by measuring reaction time differences between emotionally laden and neutral words. For example, Kirsh et al.^22^ showed that violent video gamers experienced significant interference when processing negatively valenced words, indicating a cognitive bias toward emotionally congruent stimuli. This finding aligns with the GAM principles, suggesting that violent video gaming amplifies the salience and processing of negative information^23^. However, the incidental emotional relevance of the words in Stroop tasks restricted the direct assessment of processing bias. To address this concern, Kirsh et al.^24^ conducted a follow-up study using a face-morphing paradigm. In this task, participants identified emotions as neutral faces morphed into happy or angry expressions. The results revealed that individuals with frequent violent media exposure recognized angry faces more quickly and happy faces more slowly, regardless of their baseline aggression levels. Likewise, Kirsh and Mounts^25^ found that participants briefly exposed to violent video games exhibited reduced recognition of happy expressions, reflecting an attentional bias toward aggressive emotional cues. This impaired happy-face advantage was further evidenced by reduced event-related potentials (ERPs) associated with attentional allocation to positive emotions in individuals exposed to violent media, including video games^26^.

In addition to script theory, research has shown that frequent violent media exposure can paradoxically desensitize individuals to negative emotions and related stimuli^27,28^. For instance, Bartholow et al.^29^ observed a reduced P300 in habitual violent gamers when exposed to violent images, suggesting impaired activation of the aversive motivational system. These individuals showed a blunted ability to categorize violent images. Similarly, Arriaga et al.^30^ found that frequent violent gaming led to emotional desensitization, specifically manifesting as reduced feelings of displeasure when gamers encountered violent emotional stimuli that would ordinarily elicit negative affect. Stockdale et al.^31^ extended this argument a step further by showing that violent media exposure does not merely dull responses to violence but also erodes sensitivity to positive and self-regulatory cues. They found that habitual violent video gamers showed lower empathy and reduced brain activity compared to less frequent gamers, as indicated by decreased P100 and N200/P300 amplitudes. They also showed reduced N200/P300 amplitudes during response inhibition, suggesting fewer neural resources were engaged to control behavior. A similar pattern of emotional desensitization has also been reported among individuals who frequently consume passive forms of violent media, such as television and films^32,33^.

Typically, desensitization to violence is conceptualized as a form of habituation^34^. However, several studies have demonstrated that desensitization can occur even after relatively brief violent media exposure^35^. Carnagey et al.^28^ demonstrated that a 20-minute session of violent gaming lowered heart rate and skin conductance to actual fight footage, while Fanti et al.^32^ reported analogous effects following a short violent film clip. At the neural level, Engelhardt et al.^36^ observed reduced P300 amplitudes to negative images among habitual violent gamers, suggesting weakened engagement of the aversive motivational system. Likewise, Stockdale et al.^37^ found that a brief exposure to violent film suppressed early discrimination components (i.e., N170, P200) and altered inhibitory control markers (i.e., N200, P300) during emotional face processing. Extending this line of research, Miedzobrodzka et al.^38^ found that both habitual and short-term gaming led to notable reductions in the P300 and P625 amplitudes in response to painful stimuli, suggesting a rapid onset of desensitization even among those previously unexposed to violent games.

Although studies endorse script theory to explain how violent media impacts emotional processing, their reliance on short-term exposure sessions undermines the very premise they seek to test. Script theory argues that aggressive knowledge structures are learned and reinforced through repeated, habitual encounters with violent content^35,39^, yet many experiments exposed participants to only a few minutes of gameplay or film clips before task performance^22,24,25^. While such acute priming can certainly reveal momentary biases, they fall short in addressing (a) how long these biases persist, (b) how they interact with the habitual nature of media use that individuals accumulate over the years, and (c) whether these effects reflect more than just mood-congruent priming^40^ or a transient stress-like response^41^ instead of a broader, more generalized impact of violent media exposure.

Conversely, the desensitization research posits a gradual dampening of emotions after frequent encounters with violence, yet a few ERP and fMRI studies exposed participants only for a short period of time^28,36,37,42^. These brief sessions can produce “transient habituation” that is not equivalent to the sustained emotional numbing that the desensitization framework predicts^43^. Supporting this criticism, several neuroimaging and ERP studies have failed to find evidence that exposure to violent media leads to desensitization^44–46^. Therefore, research that backs the GAM offers mixed evidence about how well its findings hold up in everyday life and over time, leaving behind a question of whether transient changes translate into real-life hostile emotional processing.

### Problematization

The present study assesses emotional processing using a facial emotion recognition paradigm, which is widely regarded as a reliable tool for examining socio-cognitive functioning. Facial emotions provide critical cues for interpersonal communication, allowing individuals to interpret emotions and adjust their behavior^47,48^. Efficient recognition of emotional expressions reflects enhanced cognitive and perceptual skills like executive functioning, sustained attention, and rapid information processing^49^. In general, it has been reported that happy expressions are often recognized more accurately than negative ones (such as anger, fear, and disgust), which is attributed to a phenomenon called the “happy-face advantage”^50^.

The GAM posits that repeated violent media exposure can erode this happy-face advantage by altering social information processing and creating attentional biases toward aggression-related emotional cues^16,17,22^. However, it is important to acknowledge that the GAM literature presents mixed results in determining whether exposure to violent media impairs facial emotion recognition, and if so, whether this impairment is emotion-specific. While some studies indicate reduced accuracy in recognizing happy faces following violent media exposure^24,25^, others report impaired recognition of negative emotions due to desensitization effects^51,52^. Additionally, an emerging body of literature documents counterintuitive or null findings^46,53,54^, thereby raising concerns about the consistency and replicability of earlier results. Although such inconsistencies are evident in the broader emotional processing literature, direct evidence within the domain of facial emotion recognition remains limited and inconclusive. Consequently, it can be premature to reject the GAM’s claim that violent media exposure reduces the happy-face advantage^24,25^. In light of this gap, we align with the GAM framework in formulating our initial hypothesis, presuming that habitual violent media users will exhibit a reduced happy-face advantage.

**H1**: The happy-face advantage will be reduced among habitual violent media users, such that recognition of happy facial emotions will be relatively less efficient than recognition of negative emotions (i.e., anger, fear, and sadness).

This hypothesis primarily tests the GAM’s assertion that violent media exposure biases emotional information processing against positive emotions such as happiness. While H1 could also be framed from an emotional desensitization perspective, doing so introduces a conceptual inconsistency. Desensitization research emphasizes a generalized blunting of affective responsiveness to emotional cues after repeated exposure to violent content^28,29,36^. Although there is evidence for reduced sensitivity to negative emotions following violent media exposure, it remains unclear whether such blunting extends to positive affective cues^51,52^. Moreover, GAM studies have seldom suggested that a reduced happy-face advantage can also be interpreted through the lens of emotional desensitization. Therefore, in our study, if happy faces are recognized more efficiently than negative emotions, the findings would reject H1 and directly challenge the GAM’s proposition that violent media exposure biases emotional information processing against positive emotions.

Furthermore, this inquiry is coupled with a critical yet underexplored issue of whether facial emotion recognition differs between interactive and non-interactive violent media users. Prior research on violent media effects has predominantly focused on single-format exposure, with limited attention given to the role of media interactivity in shaping aggression-related outcomes. Most studies have compared users of violent interactive media (i.e., video games) with either non-users or users of non-violent content^51,53,54^. This gap is theoretically significant because interactivity has been proposed as a key factor amplifying media effects^7^. Unlike passive media, video games require continuous user engagement and goal-directed activities while engaging in hostile gameplay. This active participation is theorized to enhance the encoding and reinforcement of aggressive scripts and cognitive biases^7^.

Despite theoretical assertions from the GAM^9^, research is limited in directly comparing these two modes of media exposure in terms of their effects on social information processing. To address this gap, the present study compares habitual users of interactive violent media with non-interactive media users to examine whether repeated exposure reduces the happy-face advantage and impairs the recognition of negative emotions. The interactive media group consists of habitual violent video gamers (VVGs), and the non-interactive media group comprises non-video gamers (NVGs) who regularly consume violent content through passive formats such as films and television, with no prior history of playing video games. Prior research suggests that violent video games elicit stronger aggressive outcomes than passive media formats^7,55^, suggesting that interactivity uniquely intensifies the cognitive and affective consequences of violent content. Within the GAM framework, heightened aggression is expected to compromise socio-cognitive skills, particularly the decoding of emotional expressions^9^. If interactivity amplifies aggression, it should also magnify these emotion recognition deficits. Therefore, assuming the differential effects of media interactivity, we propose the following hypothesis.

**H2**: Compared to NVGs, habitual VVGs will demonstrate reduced recognition capacities across positive and negative facial emotions, such as happiness, anger, fear, and sadness.

In addition to examining the effects of habitual violent media exposure, this study accounts for individual differences and incorporates trait aggression as a control variable. Individuals with a high baseline level of aggression may already exhibit biased emotional information processing, independent of media exposure^56^. While violent media may reinforce their actions, it is unlikely to be the sole contributor to escalated aggression. Moreover, aggression is a multifaceted construct influenced by a range of environmental and psychosocial factors, including family violence, early childhood experiences, substance abuse, and socioeconomic adversity^57–59^. Therefore, the emotional processing impairments may be partially attributable to these underlying influences rather than media exposure alone^11^. This approach allows for a more accurate assessment of whether observed deficits in facial emotion recognition are primarily related to media exposure or are confounded by dispositional aggression.

## Methods

### Participants

A power analysis was initially conducted using G*Power (version 3.1.9.7)^60^ to determine the minimum number of participants required for the experiment. The analysis indicated that a sample size of 24 participants (12 in each group) would be sufficient to detect a medium effect size (*f* = 0.25) at 0.05 alpha error probability (two-tailed) with the power of 0.80 in a repeated measures analysis of variance (ANOVA) for within-between interaction effects. Participants were recruited from a higher education institution in India through an online announcement posted on departmental mailing lists. Interested students first completed a pre-screening survey that assessed violent media consumption and gaming history. Two mutually exclusive user groups were defined a priori, consisting of habitual interactive and non-interactive violent media users. For convenience, these groups are termed VVGs and NVGs, respectively.

This study established rigorous inclusion and exclusion criteria for each group, covering the duration and intensity of media engagement and cross-modal exposure limits. VVGs were required to have played predominantly violent video games for at least two hours per day over the past 12 months. The reported games were validated based on content ratings from ESRB and PEGI. Participants with non-aligned gaming preferences were excluded. Additionally, given the likelihood of overlapping media exposure, the study assessed general violent media consumption. Those who had spent more than an hour per day on non-interactive violent media (e.g., movies, TV shows) over the past 12 months were also excluded. Of 52 volunteers who self-identified as gamers, 25 were excluded for insufficient daily playtime (*n* = 4), frequent mixed-genre play (*n* = 7), and high consumption of non-interactive violent media (*n* = 14). None of them met the threshold for gaming addiction^61,62^.

Meanwhile, NVGs comprised individuals who frequently consumed violent media, such as movies, television, web series, and documentaries, for the past 12 months, engaging a minimum of two hours per day with such content. They were also required to have abstained from all video gaming during the previous 12 months. To ensure eligibility, their past gaming history was reviewed, and those who had ever engaged in regular violent video game play were excluded. Of 46 volunteers, 11 were excluded for recent gaming involvement. 27 of the remaining 35 eligible NVGs were randomly selected to match the VVG group. The final sample comprised 54 healthy adults with an equal number of VVGs (*M*_*age*_ = 20.07 years, *SD* = 1.26) and NVGs (*M*_*age*_ = 21.29 years, *SD* = 1.10).

### Ethical statement

The study adhered to the principles outlined in the Declaration of Helsinki. Written informed consent was obtained from each participant before the experiment, including details on participation and publication consent. All participants, including those who did not participate but expressed their interest in the study, received non-monetary incentives. The study also obtained necessary approval from the “Institute Human Ethics Committee” of the authors’ affiliating institute.

### Questionnaire

#### Gaming addiction

The Internet Gaming Disorder Scale-Short-Form (IGDS9-SF)^61^ was utilized as a screening test to assess potential addiction trends among gamers. The questionnaire employed a 5-point Likert scale (1 = *never* to 5 = *very often*) to measure responses. To distinguish between addicted and non-addicted gamers, researchers examined whether participants endorsed at least five criteria out of the nine, with a particular focus on responses marked as “very often.” These responses were considered an endorsement of the specific criterion. However, no participant met these criteria, indicating that none exhibited signs of addiction.

#### Trait aggression

Trait aggression was assessed using a 29-item Buss and Perry Aggression Questionnaire (BPAQ)^63^. All participants indicated their level of agreement using a 5-point Likert scale (1 = *does not describe me at all* to 5 = *describes me very well*). The scale exhibited high internal consistency (Cronbach’s α = 0.84).

#### Violent media content

Habitual exposure to violent media was assessed using a 12-item Content-Based Media Exposure Questionnaire (C-MEQ)^64^. A sample item for the NVGs was: “How often do you watch, on movies/web series/documentaries, people who shoot at another person?” This scale was modified for gamers to specifically measure violent media exposure related to their gaming activities. The same question was adjusted to: “How often do you engage in video games shooting at another person?” The responses were given on a 5-point Likert scale (1 = *never* to 5 = *very often*), allowing participants to indicate the frequency of their exposure to violent media content. The scale demonstrated high internal consistency when used on VVGs (Cronbach’s α = 0.89) and NVGs (Cronbach’s α = 0.84).

#### Gaming details and preferences

Gamers were asked to report average daily playtime (in hours) and overall gaming experience (in years). They also rated three of their preferred violent video games played over the past 12 months using a 7-point Likert scale (1 = *rarely* to 7 = *often*). They rated each game on the frequency, perceived content of violence, and perceived severity (exposure to blood and gore). This rating scale allowed participants to express their subjective evaluation of their preferred games.

### Stimuli

A total of 320 (Male = 160, Female = 160) static facial emotions were used from the KDEF (The Karolinska Directed Emotional Faces) dataset^65^. Among those, 112 faces carried neutral expressions, and the remaining 208 carried emotional expressions (52 each). The stimulus presentation was controlled using E-Prime 3 software, which was operated on an HP Desktop PC with specifications including a 12th Gen Intel^®^ Core™ i7-12700 processor running at 2,100 MHz and equipped with 12 cores. The faces in the experiment were presented in grayscale for the forced-choice trials and with a blue tint for the free-choice trials, with a resolution of 560 × 720 pixels. These visual manipulations were employed to distinguish between the two response conditions and ensure clarity in the experimental setup.

### Research paradigm

This study employed a modified emotional go/no-go paradigm^66^. In this task, participants engaged in forced-choice trials where they were instructed to respond by pressing a designated key when a target facial emotion (go trials) was presented and to withhold their response when any non-target emotion (no-go trials) appeared. Facial emotion recognition capacity was assessed through the number of correct responses (CRs) on go trials and the corresponding reaction times (RTs). Instances where participants responded during no-go trials were counted as false alarms (FAs), which served as an indicator of response inhibition failure^67^. A higher number of CRs, faster RTs, and fewer FAs are presumed to indicate enhanced recognition and inhibitory control for specific emotional expressions. Additionally, the present study incorporated free-choice trials, offering a distinct contrast to traditional forced-choice designs^66,67^. Free-choice trials allowed participants to decide whether or not to respond without any constraints. While the forced-choice trials captured the performance of an individual with respect to the rate of CAs and FAs, free-choice trials supplemented the analysis through their “free will” framework^68^.

### Experimental procedure

All participants were assessed in a semi-dark, soundproof laboratory setting to ensure controlled environmental conditions. The go/no-go task^66^ combined emotional and neutral faces to create eight blocks of go/no-go trials (anger/neutral, neutral/anger, fear/neutral, neutral/fear, happy/neutral, neutral/happy, sad/neutral, and neutral/sad). In each pair, the first facial emotion served as the “go” trial, and the second as the “no-go” trial. In addition to the forced-choice, encompassing both go and no-go trials, free-choice trials were integrated into the protocol. These trials featured a distribution of facial emotions different from those used in the go and no-go trials, allowing participants to exercise decision-making without predefined responses.

**Fig. 1:**
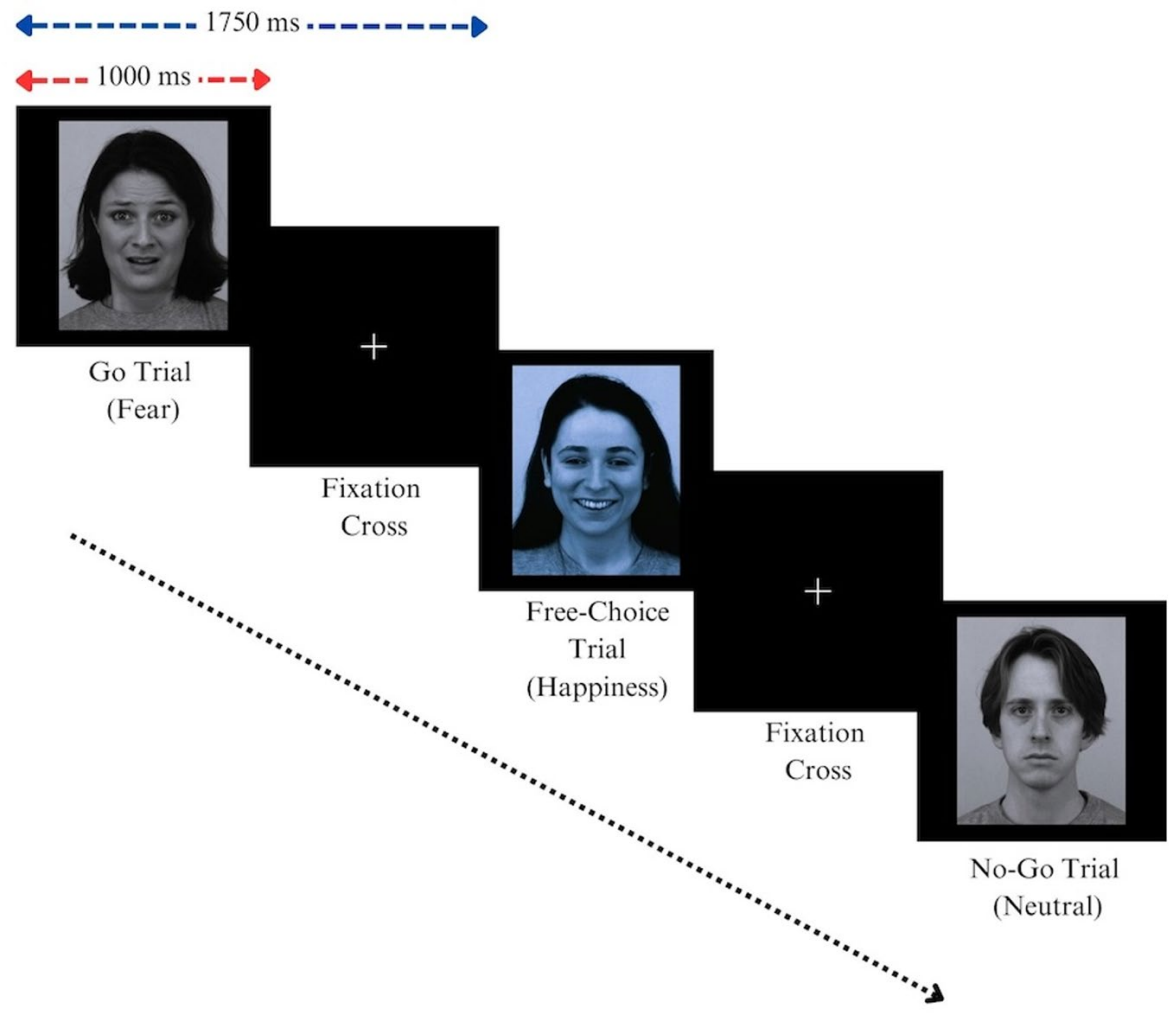
Stimulus presentation sequence in a sample Fear/Neutral block. Fearful face is designated as a “go” trial, while a neutral face serves as a “no-go” trial, and a happy face (depicted with a blue tint) represents the free-choice trial. All the representative stimuli are used with permission from the Psychology Section at Karolinska Institutet for research purposes^65^.

Each task block consisted of 40 trials, broken down into 20 go trials, 12 free-choice trials, and eight no-go trials. A prepotency was established, with go-trials comprising 50% of the total trials, highlighting their prominence in the task structure. A significant proportion (30%) of the trials were allocated as free-choice to probe the effect of participants’ free will on task performance. The experiment started with a briefing followed by a short practice block of 15 trials to understand the nature of the task. During go trials, participants were instructed to press the right arrow key on a keyboard upon seeing a designated facial emotion. Conversely, for no-go trials, participants were told to refrain from pressing the key. The specific emotional valence of no-go trials was not disclosed to prevent anticipatory strategies. During free-choice trials, participants encountered blue-tinted facial expressions (see Fig. 1) and were encouraged to rely on instinctive judgments to respond or withhold response, thus simulating a more naturalistic decision-making environment. To mitigate the effects of practice and fatigue, the sequence of task blocks was randomized. Furthermore, the trials within each block were arranged in a pseudo-randomized order to avoid more than two consecutive no-go trials. Each trial was displayed for a fixed duration of 1,000 ms, followed by a fixation cross that was shown for 750 ms. Careful measures were taken to ensure participants fully understood the instructions and were competent to perform the tasks accurately. On average, the participants completed the experimental task within 30 min.

### Study design

The study incorporated a 2 (experimental group: VVGs vs. NVGs) × 4 (facial emotion: anger vs. fear vs. happiness vs. sadness) mixed factorial design, where the first factor was a between-subjects variable.

### Data analyses

An independent samples *t*-test was conducted to analyze the differences between VVGs and NVGs on aggression and violent media exposure. In the core analyses, a 2 (experimental group: VVGs vs. NVGs) × 4 (facial emotion: anger vs. fear vs. happiness vs. sadness) two-way repeated measures ANOVA was performed on the percentage of CRs on go trials, RTs on correct go trials, the percentage of FAs on no-go trials, and the percentage of responses on free-choice trials.

The percentage of CRs and the corresponding RTs on the go trials were used to measure the accuracy of facial recognition. The percentage of FAs on no-go trials was used to measure the rate of response inhibition failure. The percentage of responses in free-choice trials provides insights into participants’ inclination toward specific emotional expressions, complementing response accuracy and inhibition measures. Further, to examine whether the group or emotion effects observed in the ANOVA persisted after accounting for trait aggression, we conducted repeated measures analyses of covariance (ANCOVA). The Greenhouse-Geisser correction is reported for *F*-statistic scores wherever Mauchly’s sphericity test was significant. Following a significant main effect of facial emotion, post hoc pairwise comparisons were conducted, and Bonferroni adjustments were applied to control for multiple comparisons.

## Results

Scores on the independent samples *t*-test showed that aggression levels were comparable across groups, *t*(52) = −0.89, *p* = 0.38, as were scores on the violent media exposure measure, *t*(52) = −1.19, *p* = 0.24. Specifically, VVGs (*M* = 66.19, *SD* = 14.83) and NVGs (*M* = 69.70, *SD* = 14.21) did not show alarming aggression levels. Gaming-related measures were also collected within the VVG group. The gaming addiction score (*M* = 20.44, *SD* = 5.96) fell below the clinical threshold^61^. VVGs reported a mean gaming experience of 7.37 years (*SD* = 3.85) and an average daily playtime of 2.29 h (*SD* = 0.46). Most of the gamers were engaged in playing *Valorant* over the past 12 months, which emerged as the top choice across the preferences, with approximately 40.71% of them expressing their first preference for the game (frequency = 5.81; perceived violence = 4.27; perceived blood and gore = 2.81). A comprehensive overview of the gaming preferences and corresponding ratings is provided in the Supplementary Material.

### Percentage of correct responses (CRs) on go trials

**Fig. 2:**
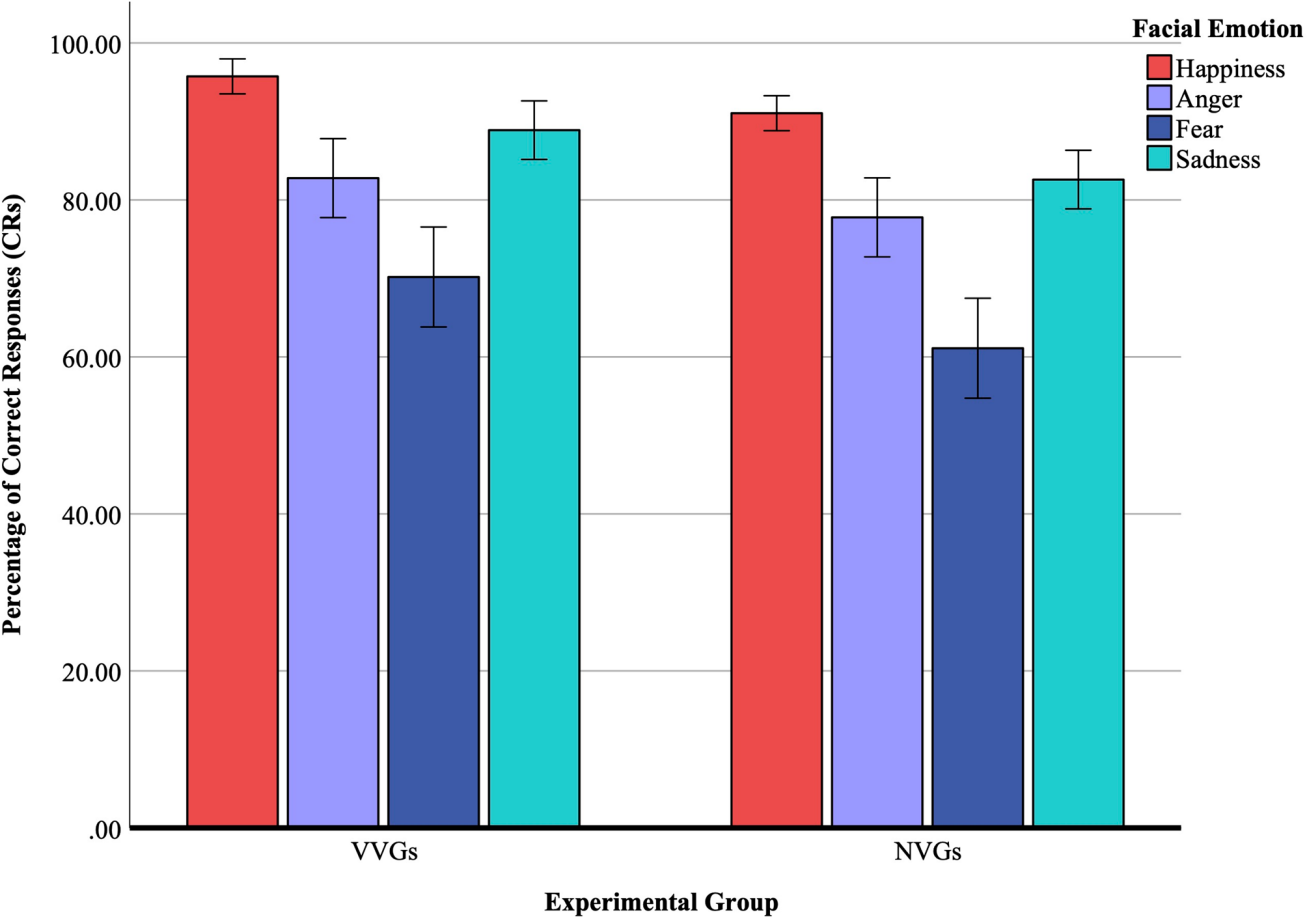
Percentage of correct responses (CRs) on go trials. This figure displays the mean percentage of CRs for happiness, anger, fear, and sadness across two experimental groups: violent video gamers (VVGs) and non-video gamers (NVGs). Error bars represent ± 2 standard errors of the mean. Both groups showed the highest recognition accuracy for happy faces. VVGs significantly outperformed NVGs across all emotion categories.

The repeated measures ANOVA revealed a main effect of the experimental group, *F*(1, 52) = 11.64, *p* < 0.001, η_p_^2^ = 0.18, indicating that the percentage of CRs was significantly higher (*p* < 0.001) in the VVGs (*M* = 84.28%, *SE* = 1.30) than in the NVGs (*M* = 78.26%, *SE* = 1.30 ). A main effect of facial emotion was observed, *F*(2.54, 132.52) = 57.02, *p* < 0.001, η_p_^2^ = 0.52. Post hoc pairwise comparisons showed that the percentage of CRs was significantly higher for happiness (*M* = 93.39%, *SE* = 0.78), when compared with anger (*M*_*difference*_ = 13.12%, *SE* = 1.99, *p* < 0.001), fear (*M*_*difference*_ = 27.75%, *SE* = 2.36, *p* < 0.001), and sadness (*M*_*difference*_ = 7.66%, *SE* = 1.62, *p* < 0.001). There was no interaction effect between the experimental group and facial emotion, *F*(2.54, 132.52) = 0.41, *p* = 0.74, η_p_^2^ = 0.01. Results are further illustrated in Fig. 2.

An ANCOVA was conducted to examine the effects of group and emotion on CRs, while statistically controlling for trait aggression. In the ANCOVA, the effect of trait aggression on CR was not significant, *F*(1, 51) = 1.18, *p* = 0.28, η_p_^2^ = 0.02. However, controlling for trait aggression maintained the group effect, *F*(1, 51) = 10.63, *p* < 0.001, η_p_^2^ = 0.17, but the emotion effect was no longer significant, *F*(2.56, 130.72) = 0.76, *p* = 0.50, η_p_^2^ = 0.02. The interaction effects, emotion × aggression, *F*(2.56, 130.72) = 0.57, *p* = 0.63, η_p_^2^ = 0.01, and emotion × group, *F*(2.56, 130.72) = 0.46, *p* = 0.82, η_p_^2^ = 0.006 were also nonsignificant. Although aggression itself was not associated with CR performance, its inclusion altered the partitioning of variance, inflating the error term for the emotion factor and eliminating its significance. The variability in aggression overlapped with emotion-specific variance, but not to a degree that significantly alters overall CR performance. These results suggest that the accuracy advantage of VVGs is substantial, whereas the differential ease of recognizing specific emotions is sensitive to individual differences in aggression.

### Reaction times (RTs) for correct go trials

**Fig. 3:**
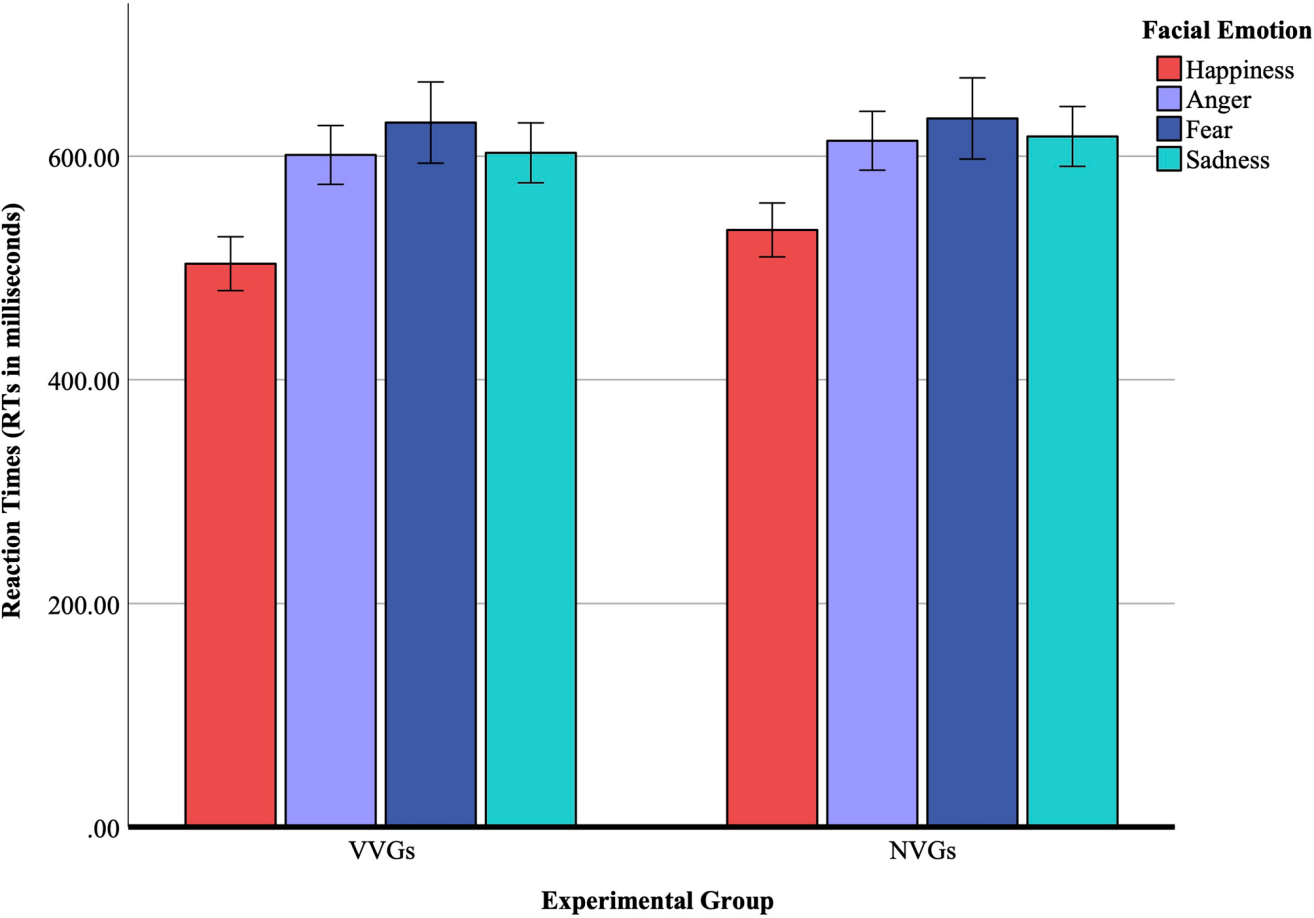
Reaction times (RTs) for correct go trials. This figure displays the mean reaction times (RTs in milliseconds) for recognizing happiness, anger, fear, and sadness across two experimental groups: violent video gamers (VVGs) and non-video gamers (NVGs). Error bars indicate ± 2 standard errors of the mean. Both groups responded most quickly to happy faces and most slowly to fearful ones. No significant group differences were found.

There was no main effect of the experimental group, *F*(1, 52) = 0.97, *p* = 0.32, η_p_^2^ = 0.02, indicating that the RTs for correct go trials were comparable between VVGs (*M* = 584.53 ms, *SE* = 10.97) and NVGs (*M* = 599.84 ms, *SE* = 10.97). A main effect of facial emotion was observed, *F*(2.53, 131.95) = 43.33, *p* < 0.001, η_p_^2^ = 0.45. Post hoc pairwise comparisons showed that the RTs were significantly shorter for happiness (*M* = 518.92 ms, *SE* = 8.52), when compared with anger (*M*_*difference*_ = −88.58 ms, *SE* = 9.89, *p* < 0.001), fear (*M*_*difference*_ = −113.03 ms, *SE* = 12.49, *p* < 0.001), and sadness (*M*_*difference*_ = −91.44 ms, *SE* = 8.02, *p* < 0.001). There was no significant interaction effect between the experimental group and facial emotion, *F*(2.53, 131.95) = 0.52, *p* = 0.66, η_p_^2^ = 0.01. Results are further illustrated in Fig. 3.

The ANCOVA yielded no significant covariate effect, *F*(1, 51) = 0.01, *p* = 0.91, η_p_^2^ = 0.001. After adjustment, the group main effect remained nonsignificant, *F*(1, 51) = 0.97, *p* = 0.33, ηp² = 0.02, and the emotion main effect was no longer significant, *F*(2.57, 131.07) = 1.86, *p* = 0.15, η_p_^2^ = 0.04. Additionally, the interaction effects, emotion × aggression, *F*(2.57, 131.07) = 1.51, *p* = 0.22, η_p_^2^ = 0.03, and emotion × group, *F*(2.57, 131.07) = 0.68, *p* = 0.55, η_p_^2^ = 0.01, were also nonsignificant. Thus, individual differences in trait aggression statistically accounted for the previously observed emotion-based RT differences, whereas gaming status had no measurable influence on the speed of facial emotion recognition.

### Percentage of false alarms (FAs) on no-go trials

**Fig. 4:**
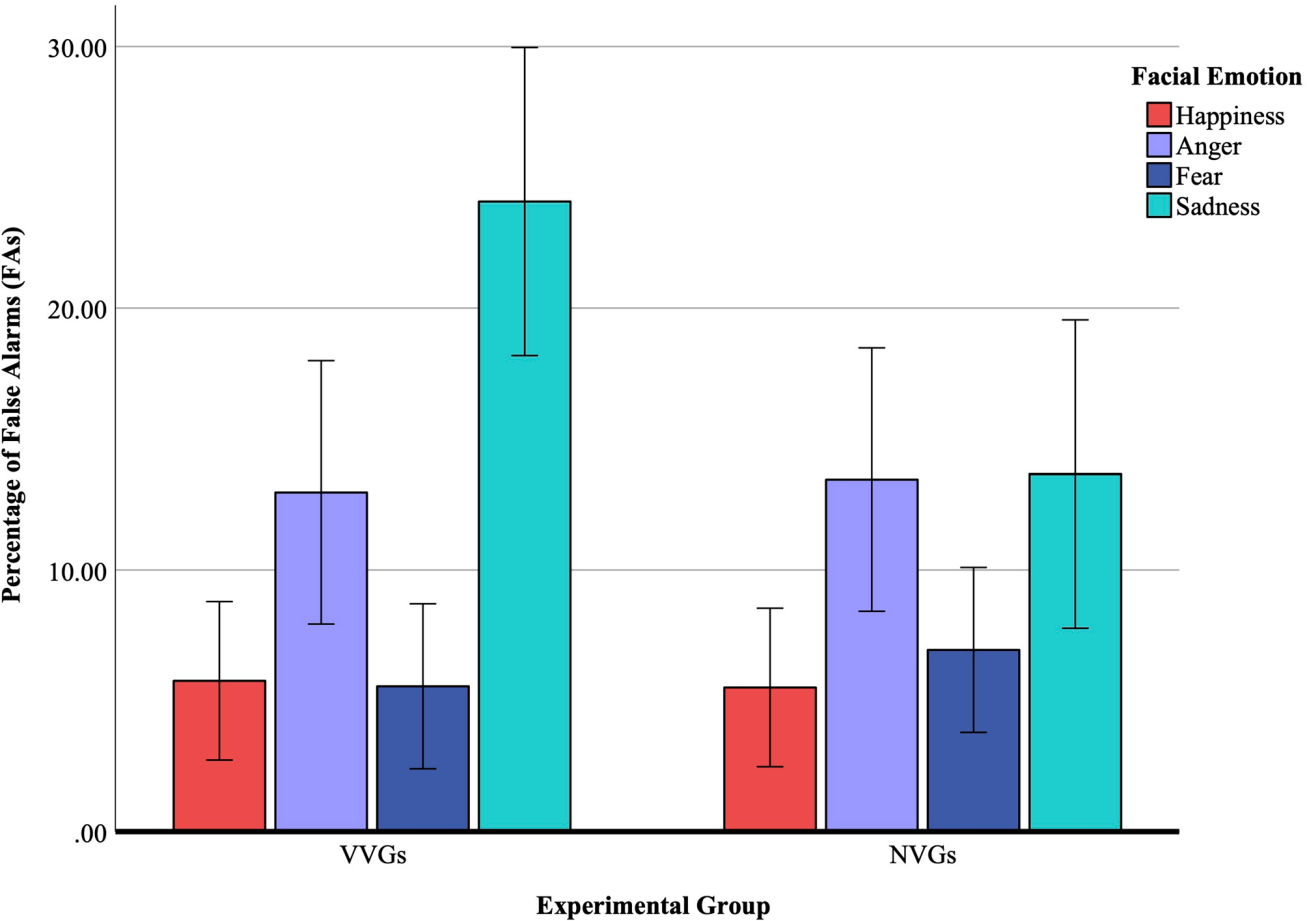
Percentage of false alarms (FAs) on no-go trials. This figure displays response inhibition rates, indicated by the percentage of FAs for recognizing happiness, anger, fear, and sadness across two experimental groups: violent video gamers (VVGs) and non-video gamers (NVGs). Error bars indicate ± 2 standard errors of the mean. Response inhibition was highest for sad faces and lowest for happy and fearful faces, with no significant differences observed between groups.

There was no main effect of the experimental group, *F*(1, 52) = 1.43, *p* = 0.23, η_p_^2^ = 0.03, indicating that the percentage of FAs for no-go trials was comparable between VVGs (*M* = 12.09%, *SE* = 1.30) and NVGs (*M* = 9.89%, *SE* = 1.30). The main effect of facial emotion, *F*(2.56,133.60) = 18.13, *p* < 0.001, η_p_^2^ = 0.25, with the post hoc pairwise comparisons, showed that the percentage of FAs was significantly higher (*p* < 0.001) for sadness (*M* = 18.87%, *SE* = 2.08). Although the percentage of FAs for happiness was the lowest, it did not differ significantly from fear (*M*_*difference*_ = −0.61%, *SE* = 1.54, *p* = 1.00), but was significantly lower than anger (*M*_*difference*_ = −7.57%, *SE* = 2.05, *p* < 0.001) and sadness (*M*_*difference*_ = −13.23%, *SE* = 2.42, *p* < 0.001). Results are further illustrated in Fig. 4.

Again, the ANCOVA yielded no significant covariate effect, *F*(1, 51) = 0.28, *p* = 0.60, η_p_^2^ = 0.001. There was no group main effect, *F*(1, 51) = 1.24, *p* = 0.27, η_p_^2^ = 0.02, neither there was emotion main effect, *F*(2.58, 131.47) = 2.68, *p* = 0.06, η_p_^2^ = 0.05. Although, the emotion × aggression effect, *F*(2.58, 131.47) = 0.78, *p* = 0.49, η_p_^2^ = 0.02, was nonsignificant, the emotion × group effect, *F*(2.58, 131.47) = 3.05, *p* = 0.04, η_p_^2^ = 0.06, appeared significant. Thus, group-specific difficulties inhibiting responses to sad expressions persisted independently of aggression, whereas general emotion-based differences in response inhibition partially overlapped with individual variability in trait aggression.

### Free-choice trials

**Fig. 5:**
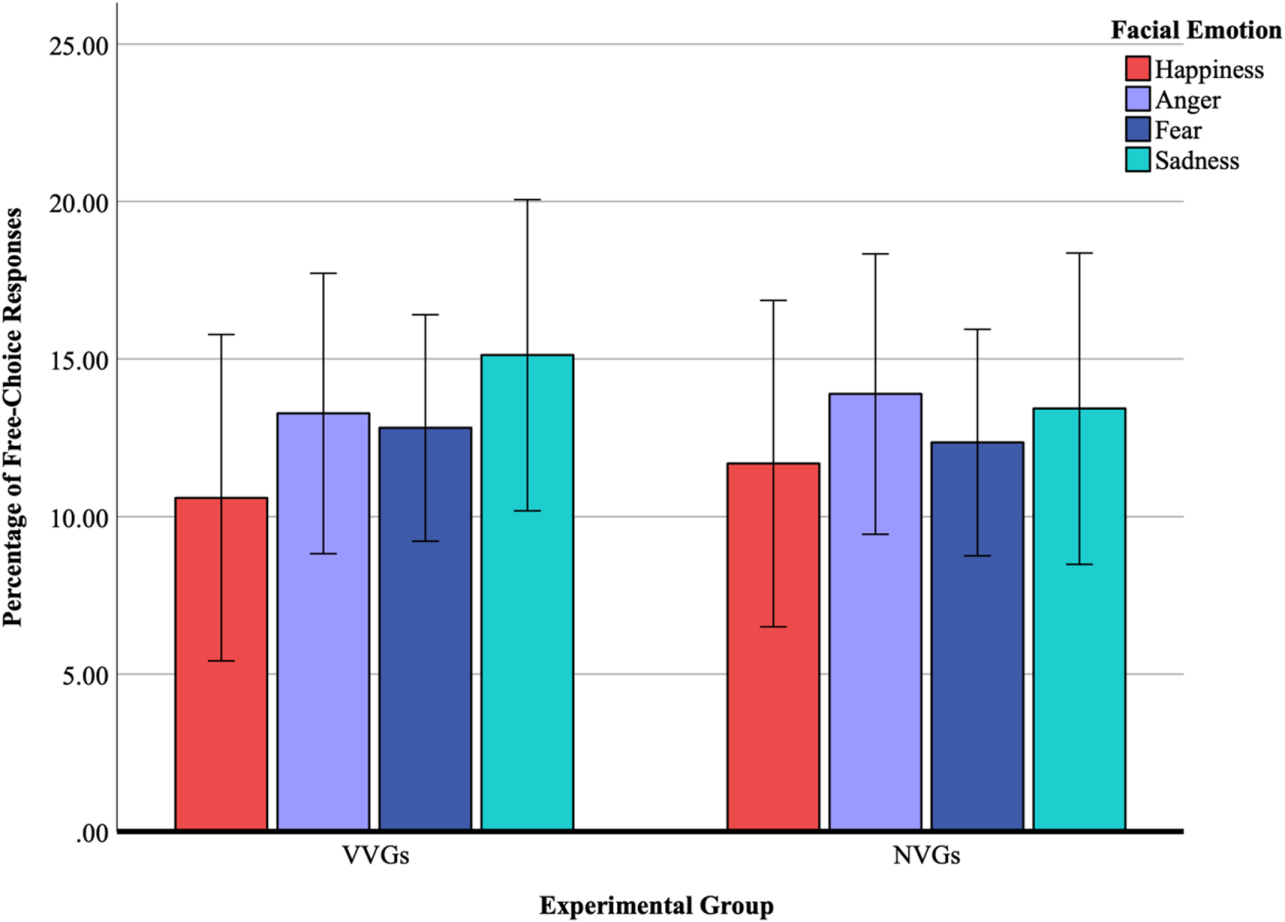
Percentage of free-choice responses. This figure displays the percentage of free-choice responses for recognizing happiness, anger, fear, and sadness across two experimental groups: violent video gamers (VVGs) and non-video gamers (NVGs). Error bars indicate ± 2 standard errors of the mean. No significant group differences or emotion-specific preferences were observed.

There was no main effect of the experimental group, *F*(1, 52) = 0.002, *p* = 0.96, η_p_^2^ = 0.001, indicating that the response rates for the free-choice trials were comparable between VVGs (*M* = 12.95%, *SE* = 1.81) and NVGs (*M* = 12.83%, *SE* = 1.81). There was no main effect of the facial emotion, *F* (2.37,123.52) = 1.41, *p* = 0.24, η_p_^2^ = 0.02. Also, there was no significant interaction effect of the experimental group and facial emotion, *F*(2.37,123.52) = 0.29, *p* = 0.78, η_p_^2^ = 0.06. Results are further illustrated in Fig. 5.

The ANCOVA yielded no significant covariate effect, *F*(1, 51) = 2.41, *p* = 0.13, η_p_^2^ = 0.05. After adjustment, the group effect remained nonsignificant, *F*(1, 51) = 0.02, *p* = 0.89, η_p_^2^ = 0.001, so as the emotion main effect, *F*(2.31, 117.69) = 2.13, *p* = 0.10, η_p_^2^ = 0.04. The interaction effects, emotion × aggression, *F*(2.31, 117.69) = 1.83, *p* = 0.16, η_p_^2^ = 0.04, and emotion × group, *F*(2.31, 117.69) = 0.33, *p* = 0.75, η_p_^2^ = 0.007, were also nonsignificant Interestingly, a quadratic contrast for facial emotion was marginally significant, *F*(1, 51) = 4.76, *p* = 0.03, suggesting slightly more errors for anger and sadness than for happiness, but this trend did not survive the omnibus test. Overall, free-choice trial accuracy did not differ between VVGs and NVGs nor across facial emotions, and these patterns were unaffected by individual differences in aggression.

## Discussion

This study addressed two key research questions on the effects of habitual violent media exposure. First, it examined whether such exposure increases aggression, thereby biasing emotional information processing, measured through a facial emotion recognition task. Second, it explored whether recognition impairments (if there are any) vary based on the mode of interaction with violent media. The study also investigated the influence of trait aggression on facial emotion recognition performance.

The results rejected H1, as happy faces were recognized more efficiently than negative emotions. This happy-face advantage was consistent across all the examined performance metrics. The results also rejected H2 as VVGs performed comparably to NVGs in terms of RTs and FAs, but outperformed NVGs in CRs. However, the free-choice trials did not yield any significant results. While trait aggression did not significantly predict performance on any outcome variable, its inclusion as a covariate in the ANCOVA consistently eliminated the statistical significance of emotional effects previously observed in the ANOVA. This pattern highlights the methodological importance of accounting for dispositional traits when studying media effects. It also reinforces the view that media exposure does not act independently but interacts with individual predispositions to shape emotional and cognitive outcomes.

We assessed facial emotion recognition performance using forced-choice and free-choice trials within an emotional go/no-go paradigm. In forced-choice trials, the happy-face advantage was evident, characterized by higher CR rates, faster RTs, and fewer FAs while recognizing happy emotions. This pattern has been consistently reported in recent literature^69–71^. Additionally, incorporating free-choice trials allowed for an exploration of emotion processing under endogenous inhibition, aligning the paradigm more closely with naturalistic emotional control^72,73^. In classical go/no-go paradigms, inhibition is externally signaled, requiring participants to withhold responses based on external cues^66^. However, real-life inhibitory control frequently involves voluntary decision-making, or free will. Schel and Crone^68^ introduced the usage of the free will paradigm to the emotional go/no-go task, where they demonstrated that adolescents exhibited significant variability in response inhibition to emotional cues, underscoring the emotional modulation of self-initiated inhibitory control. The inclusion of free-choice trials in our study was inspired by this school of thought, which explored endogenous inhibition and voluntary action. This approach enabled us to examine individuals’ emotional orientation driven by free will rather than external cues. Although the free-choice trials did not yield definitive results, they expanded the scope of the emotional go/no-go paradigm by moving beyond traditional measures of response accuracy and inhibition to encompass voluntary emotional decision-making. Therefore, we reject the H2 only based on the forced-choice performance metrics such as CRs, RTs, and FAs.

Several complementary mechanisms help explain the robust happy-face advantage observed in this study. Firstly, happy faces are frequently available in interpersonal communications and tend to have universally recognizable features that transcend cultural boundaries^74,75^. In terms of stimulus properties, the high-contrast, curvilinear features of the smiling mouth rapidly capture overt attention. Eye-tracking studies have shown that gaze lands on and dwells longer in the mouth region of happy faces than on diagnostic regions of negative expressions^47,70,71^. Computational and behavioral work further indicates that happy faces elicit more holistic configural processing, allowing viewers to encode the expression with fewer fixation cycles^76^. On the other hand, negative emotions distribute their diagnostic cues across multiple upper facial regions, diluting perceptual salience and increasing confusability among anger, fear, and sadness^70^. Meta-analytic reviews consistently confirm that recognition accuracy for happiness exceeds that for any negative emotion^75^. Our results converge with this line of inquiry, as the recognition capacity for happy faces was superior compared to the negative ones.

Overall results suggest that the happy-face advantage remains evident among habitual violent media users, and this advantage is similar across two modes of interaction. Despite repeated exposure to violent content, their emotional recognition appears intact, with no evidence of biased processing against positive emotions as previously reported^24,25^. Importantly, the absence of such bias does not validate alternative accounts such as desensitization. Desensitization theories predict a generalized dampening of responsiveness to emotional cues, leading to reduced sensitivity for both positive and negative emotions^27–29^. In contrast, the present findings revealed reduced recognition for negative emotions “alongside” a robust happy-face advantage, a profile that aligns more closely with broader research demonstrating superior recognizability of happiness compared to negative emotions^50,70,71,75,77^. Thus, rather than reflecting desensitization, the outcomes suggest that negative emotions are inherently harder to decode, and violent media exposure did not override this baseline asymmetry in emotion processing. Taken together, these results indicate that violent media use neither supports the GAM’s prediction of biased processing against happiness nor straightforwardly maps onto a desensitization framework, but instead reflects more fundamental asymmetries in emotion recognition.

Our findings thus challenge earlier studies reporting impaired facial emotion recognition following violent media exposure, suggesting that these deficits may be less pervasive or more contingent on experimental context than previously assumed^24–26^. Previous research frequently interpreted reduced recognition of happy expressions as evidence of aggressive biases in social information processing^22^. However, many of these studies employed immediate post-exposure assessments, creating conditions that could trigger brief priming or recency effects. Such immediate testing environments are likely to exaggerate negative emotional biases temporarily, rather than represent stable, generalized cognitive shifts in everyday emotional processing^78,79^. Indeed, the influence of violent media on aggressive cognition and affect appears transient, typically subsiding within minutes after exposure ceases^80,81^.

Large-scale, preregistered longitudinal investigations have consistently failed to identify meaningful causal links between habitual violent media use and subsequent aggression or diminished prosocial behaviors^82,83^. Additionally, re-analyses of the Anderson et al.^84^ meta-analysis show that once publication bias is corrected, laboratory effect sizes shrink to the point of triviality^15^. Hilgard et al.’s^15^ analysis specifically emphasized that methodological factors and selective reporting have artificially inflated the perceived impact of violent media on aggressive outcomes. Our findings closely align with these recent critiques. In particular, our use of an ANCOVA controlling for trait aggression highlights that individual differences in baseline aggression, rather than exposure to violent media itself, largely account for the minor variations in emotion recognition performance observed. This result reinforces growing evidence that aggressive tendencies and impaired emotional recognition are influenced more robustly by pre-existing dispositional and environmental factors (e.g., developmental adversity, trait hostility, family conflict) than by media exposure per se^85–88^. Therefore, the link between violent media and impaired emotion recognition, often used as a proxy for aggression, appears neither robust nor enduring, but instead highly context-dependent and sensitive to methodological artifacts.

Furthermore, participants who predominantly engaged in interactive violent media (VVGs) consistently outperformed those exposed primarily to non-interactive violent media (NVGs) across all assessed metrics, with statistically significant advantages emerging for CRs. Given that both groups were matched for trait aggression, overall frequency, and intensity of violent media exposure, the superior performance of VVGs likely reflects cognitive and perceptual benefits specifically associated with video gaming. These findings complement previous studies suggesting a cognitive advantage among gamers in emotion recognition tasks. For instance, Ciobanu et al.^89^ found that VVGs exhibited superior performance in identifying facial expressions, primarily due to enhanced selective attention toward relevant emotional cues rather than merely reduced distractibility. This reflects greater top-down attentional control, allowing gamers to prioritize emotionally salient stimuli while effectively filtering out irrelevant information. Such attentional advantages are likely rooted in the cognitively demanding nature of violent video games, which often belong to the action genre. These games typically involve fast-paced gameplay, rapid decision-making, precise hand-eye coordination, and combat scenarios that demand quick reflexes^90^. They require players to monitor multiple dynamic elements simultaneously, thereby engaging attentional control, working memory, and executive functions^91–93^.

Taken together, our findings suggest that the superior facial emotion recognition observed in VVGs may reflect not only efficient emotional processing but also enhanced cognitive functioning. Thus, the benefits of violent video games appear to stem more from their gameplay mechanics than their thematic content^7^. Consequently, it may be misguided to critique violent video games solely on the basis of their violent themes, as they may confer meaningful cognitive and emotional advantages. Combined with evidence that violent video games train selective attention and executive control, our results suggest that any transient priming of aggressive schemas is quickly outweighed by the cognitive benefits of sustained gameplay. Although NVGs performed slightly lower than VVGs in emotion recognition, this should not be framed as an “impairment” but rather as a relative difference in efficiency.

### Limitations and future directions

Despite its intent to examine whether habitual exposure to violent media influences facial emotion recognition, this study is not without limitations. Firstly, although our findings are counterintuitive to key predictions of the GAM, a few observations temper and caution for overstated conclusions. First, the distribution of trait aggression in our sample was relatively restricted and comparable across VVGs and NVGs. Prior work indicates that emotional processing impairments and hostile attribution biases are more pronounced among individuals with high or clinically relevant aggression levels^36^. Consistent with that literature, our ANCOVA showed that even modest variability in self-reported aggression altered the statistical significance of emotion effects, suggesting that a wider aggression range might reveal deficits masked here. Accordingly, the present results should not be generalized to populations with elevated aggression or other negative dispositions.

A key concern also lies in the selection of experimental groups. Participants were recruited based on an interest survey, which introduces the possibility of self-selection bias. This may limit the external validity of the findings, as individuals who volunteer for such studies may systematically differ from the broader population. While the use of a targeted sample aligns with the study’s aims (i.e., habitual VVGs and NVGs), a more randomized sampling strategy would have enhanced generalizability and reduced the risk of sampling bias. Additionally, we acknowledge the difficulty of completely disentangling interactive from non-interactive violent media exposure. While the study attempted to isolate habitual gamers from those with frequent passive violent media exposure, such a separation is inherently challenging. Many participants may have encountered violent content on television or in online videos “incidentally” over time. This exposure could have contributed as a confounding variable. Although individuals reporting frequent passive exposure (i.e., more than one hour per day) were excluded, residual overlap cannot be ruled out. While it was relatively straightforward to identify non-gaming participants with minimal (or no) exposure to video games, it proved substantially harder to recruit gamers who had no meaningful exposure to non-interactive violent media. Given this difficulty, we recommend that future studies employ rigorous media history assessments and clearer operational definitions when it comes to media interactivity.

Another important limitation concerns the reliance on a self-report measure to assess trait aggression. Although the instrument demonstrated strong internal consistency and is widely used in aggression research, self-reports are vulnerable to social-desirability bias and have limited introspective accuracy. Such biases could have attenuated the true association between aggression and outcome variables and potentially obscured meaningful effects (e.g., the disappearance of the emotion main effect for CRs and RTs). Also, while trait aggression was considered as a covariate in the repeated measures analysis, the assumption of linearity between aggression and performance metrics was not supported. As such, ANCOVA results should be interpreted with caution. Alternatively, the null finding may also reflect the insensitivity of self-report instruments to context-specific expressions of aggression, rather than a genuine absence of behavioral or neurocognitive associations. Therefore, the disappearance of emotion effects after covarying for aggression should not lead to the premature conclusion that emotional differences are irrelevant. Even if the adjusted *p*-value exceeds 0.05, a subtle but real advantage in recognizing happiness over fear may still influence everyday social interactions. We strongly suggest that statistical adjustment should not be conflated with theoretical insignificance.

More critically, the cross-sectional nature of the study precludes any causal inference regarding the relationship between violent media exposure and aggression. Importantly, statistically controlling for aggression (i.e., ANCOVA) is not equivalent to experimental manipulation or temporal separation. Therefore, the present findings can only suggest that frequent and habitual exposure to violent media does not appear to create an aggression-related emotional information processing. In this sense, the influence of violent media may not extend to real-life perceptual or cognitive distortions in emotion recognition. Nonetheless, to address these limitations, future research should incorporate diverse assessments of aggression (e.g., behavioral tasks, peer reports, physiological indicators) and adopt designs or experimental manipulations capable of clarifying the directionality and causal mechanisms underlying the relationship between aggression and violent media exposure. We also recommend that future studies employ larger sample sizes to better test potential moderation effects of dispositional traits such as aggression. While our G*Power analysis indicates that the current sample was adequate for the primary effects examined, it may not provide sufficient power for complex analyses.

Furthermore, this study had a restricted demographic profile as most participants were male and fell within a narrow age range. Future research should incorporate more diverse samples across age and gender to explore how these factors may moderate the psychological impact of violent media. Prior work has shown that age may influence the relationship between violent video game exposure and aggressive behavior, with younger individuals potentially being more susceptible to such effects^94^. Although our results contrasted with these findings, incorporating age-wise comparisons in future research could yield more tailored inferences. Additionally, while game content was validated using ESRB and PEGI ratings, many participants reported playing titles featuring stylized or “cartoonish” violence, which can reduce the perceived realism and psychological impact. We also did not account for gameplay behaviors or customizable settings that modulate violent content. For instance, games like *Valorant* emphasize strategy and coordination over violence, and players can disable blood effects. These factors can attenuate the internalization of violent themes and should be considered in future studies examining media violence and emotional processing.

## Conclusion

Taken together, the present study offers a counterintuitive narrative on the ongoing debate about whether violent media exposure would result in aggressive information processing. Contrary to the core assumptions of the GAM, habitual exposure to violent media did not impair emotional information processing. All the participants, irrespective of the media interactivity, showed sustained happy-face advantage and conventional recognition capacity for negative emotions. Interestingly, gamers demonstrated superior emotion recognition performance, suggesting potential cognitive and attentional advantages conferred by sustained gameplay. This advantage persisted despite comparable aggression levels and media exposure across groups, pointing to gameplay mechanics, rather than violent content per se, as the likely source of performance differences. Importantly, while trait aggression did not independently predict emotion recognition outcomes, its inclusion as a covariate revealed subtle shifts in statistical significance, particularly for emotion effects. This underscores the critical role of individual dispositions in shaping media-related outcomes and highlights the importance of accounting for such variables in future research. Rather than reinforcing a simplistic causal link between violent media and emotional dysfunction, the findings support a more context-dependent and interactionist view. Although the results challenge the universality of aggression-related media effects, they do not dismiss the possibility that violent media may affect subsets of individuals, particularly those with elevated aggression.

## Supplementary Information

Below is the link to the electronic supplementary material.

Supplementary Material 1

## Data Availability

The study data are provided in the Supplementary Material.
